# Association between PARP-1 V762A Polymorphism and Breast Cancer Susceptibility in Saudi Population

**DOI:** 10.1371/journal.pone.0085541

**Published:** 2013-12-31

**Authors:** Mohammad Alanazi, Akbar Ali Khan Pathan, Zainul Arifeen, Jilani P. Shaik, Huda A. Alabdulkarim, Abdelhabib Semlali, Mohammad D. Bazzi, Narasimha Reddy Parine

**Affiliations:** 1 Genome research chair, Department of Biochemistry, College of Science, King Saud University, Riyadh, Kingdom of Saudi Arabia; 2 Medical Genetics Department, Science and Technology Unit. Umm Al Qura University. Mecca, Saudi Arabia; 3 The Comprehensive Cancer Center at King Fahad Medical City, Riyadh, Saudi Arabia; Nazarbayev University, Kazakhstan

## Abstract

Genetic aberrations of DNA repair enzymes are known to be common events and to be associated with different cancer entities. Aim of the following study was to analyze the genetic association of rs1136410 (Val762Ala) in PARP1 gene with the risk of breast cancer using genotypic assays and *insilico* structural predictions. Genotypic analysis of individual locus showed statistically significant association of Val762Ala with increased susceptibility to breast cancer. Protein structural analysis was performed with Val762Ala variant allele and compared with the predicted native protein structure. Protein prediction analysis showed that this nsSNP may cause changes in the protein structure and it is associated with the disease. In addition to the native and mutant 3D structures of PARP1 were also analyzed using solvent accessibility models for further protein stability confirmation. Taken together, this the first study that confirmed Val762Ala variant has functional effect and structural impact on the PARP1 and may play an important role in breast cancer progression in Saudi population.

## Introduction

Breast cancer is the most common neoplasm and the second leading cause of cancer death in Saudi women [1]. The recent increase in incidence has made breast cancer one of the most frequently recorded diseases among Saudi women [2,3]. The age-adjusted death rate because of breast cancer in Saudi Arabia is also rising, with the most rapid increase in the world from 1985 to 2008 [4]. This malignancy signifies a diverse group of tumors with characteristic molecular features, prognosis and responses to available therapy [5]. 

DNA repair pathways exist in each and every organism for maintaining genome integrity [6], and mutations in DNA repair pathways can result in cancer [7]. Interindividual variations in DNA repair pathways and their mechanisms have been associated with an enhanced risk of cancers [8,9]. Poly (ADP-ribose) polymerase 1 (PARP-1) is a DNA double strand break recognizing protein, and its activation is one of the early responses to DNA damage [10]. PARP-1 gene consists of 23 exons and spans about 47.3 kb which is localized on chromosome 1q41–42 [10,11]. PARP-1 catalyzes poly(ADP-ribosyl)ation, an quick DNA-damage dependent post-translational modification of itself, histones and other nuclear proteins, which is assumed to play a multifunctional role in various cellular mechanisms, including DNA-damage recognition and repair, cell death pathways and mitotic apparatus function [11,12]. PARP-1 deficiency in mice resulted in spontaneous mammary carcinomas, and additional p53 mutations shorten the latency of mammary tumor formation suggesting a possible involvement of PARP-1 in breast carcinogenesis [13]. PARP-1 role has been implicated in tumorigenesis [14,15]. Few studies indicate that PARP-1 plays a vital role in suppressing malignancy in mice. Interestingly, reduced PARP-1 activity in human peripheral blood lymphocytes has been linked with human breast, colon, lung [16] and laryngeal cancers [17].

There are several single nucleotide polymorphism's (SNP) which are reported in the PARP-1 gene, and few are reported to be associated in carcinogenesis [11,18-21]. Several SNPs have been found in PARP1 gene [22], but only for the Val762Ala (rs1136410) a functional analysis has been performed. PARP-1 V762A is a base T to C transition at codon 762 in exon 17, which results in the substitution of alanine for valine in the catalytic domain of PARP-1, and PARP-1 V762A polymorphism was reported to be associated with an altered activity of PARP-1 [19,23,24]. This amino acid substitution is responsible for a reduced activity of PARP1, thus being potentially correlated to an increased risk of disease [23]. PARP-1 Val762Ala was well-known to be associated with increased risk of several cancers [11,19,20,23]. Three studies have reported positive associations between the Val762 SNP and lung [20], esophagous [21] and prostate cancer [19]. 

To the best of our knowledge, till now there are no reports about the association between the SNPs of PARP-1 Val762Ala and breast cancer in Saudi population and there are no published reports on the structural prediction of this SNP. Together, this is the first report which deals with the association and structural studies on rs1136410 (Val762Ala) using TaqMan assays and *in-silico* studies. 

## Materials and Methods

### Study population

A total of 195 blood samples were obtained from King Khalid University Hospital. These encompassed 99 patients with cardiovascular disease and 96 healthy controls. All controls were age-matched and recruited from physical examinations after diagnostic exclusion of cancer and cancer- related diseases. Blood samples of the experimental and control groups were obtained before treatment. For gene expression studies breast cancer tissues (n=86) and normal tissues (n=40) were collected immediately after excision during surgery and stored at –80°C until use. Histopathology and medical records were reviewed to confirm diagnosis. Routine pathological variables including age, tumor grading, tumor staging and immunohistochemical determination of Estrogen Receptor (ER), Progesterone Receptor (PR) and Human Epidermal growth factor Receptor (HER2) status are illustrated in Table 1. Controls were frequency matched to cases on age/race and recruited from the clinic population receiving routine mammography at the Breast Screening and Diagnostic Center. Eligibility criteria for controls included normal mammography results and no prior cancer history. Written informed consent was obtained from all participants, and approval was received from the King Khalid University Hospital ethics review committee. Every study participant completed a self-administered baseline questionnaire, which included information on demographics, reproductive history, medical conditions and family history of cancer. 

**Table 1:** Clinical Characteristics of Study Subjects.

**Variable**	**Character**	**No of Samples**
Age (Years)	Median age	48
Estrogen receptor	ER+	53
	ER-	43
Progesterone receptor	PR+	49
	PR-	41
HER Status	HER+	38
	HER-	52
TNM staging		
	1	12
	2	35
	3	23
	4	16
Tumor grade		
	I	22
	II	35
	III	29

### DNA extraction

Approximately 3 ml of blood was collected in sterile tubes containing ethylenediaminetetraacetic acid (EDTA) from all subjects enrolled in the study. Genomic DNA was isolated from blood samples using the QIAmp kit (QIAmp DNA Blood Mini Kit, Qiagen, Valencia, CA, USA) according to the manufacturer’s instructions. After extraction and purification, the DNA was quantified using a NanoDrop 8000spectrophotometer to determine its concentration, and its purity was examined using standard A260/A280 and A260/A230 ratios (NanoDrop 8000, Thermo Scientific).

### Genotyping

SNP rs1136410 (Val762Ala) in the PARP1 gene is genotyped using a TaqMan allelic discrimination assay [25]. For each sample, 20 ng of DNA was used per reaction with 5.6 µL of 2X Universal Master Mix and 200 nM primers (Applied Biosystems, Foster City, CA, USA). All genotypes were determined by endpoint reading on an ABI 7500 real-time PCR machine (Applied Biosystems, Foster City, CA, USA). The primer and probe mixtures were purchased from the assays-on-demand service of Applied Biosystems. Five percent of the samples were randomly selected and subjected to repeat analysis as a quality control measure for verifying genotyping procedures. 

### Molecular dynamics simulation

The molecular dynamics (MD) simulations were carried out as explained by Khan et al. [26]. CHARMM (Chemistry at HARvard Macromolecular Mechanics) [27,28] and GROMACS 4.0.5 were used [29] to perform MD simulations using 5-fs time, 400 K temperature, 1 atm constant pressure and below periodic solvent boundary conditions. The simulations were performed using energy minimization and molecular dynamics to optimize predicted protein structure as well as to simulate its natural motion. The 3D structure optimization was performed in the native and mutated protein structures based on their RMSD values. Gromacs was used for force field energy minimization; conjugate gradient and limited reminiscence Broyden–Fletcher–Goldfarb–Shanno (L-BFGS) method was for solving nonlinear optimization problems. KoBaMIN program [30,31] was used to refine the predicted CRAPD structure; however the solvated system was neither minimized nor equilibrated. The free energy simulations were performed with solvent water molecules in close proximity to the solute to get an efficient solvent boundary potential (SSBP). The ion configuration based on van der Waals (vdW) interactions was verified by Monte Carlo (MC) simulation. The Particle Mesh Ewald methodology [32] was utilized for electrostatic interaction using 13 Ǻ cut-off for van der walls interactions. KCl (51 M) ions were added in the simulation box to neutralize the overall negative charge of the structures. The wild and mutated 3D structures were analyzed by CCP4 (QtMG) [28] and NOMAD-Ref was used for energy minimization [33].

### Protein structure modeling of mutant PARP1 and comparing it with the mutant

The dbSNP was utilized to identify the mutant residue location within the PARP1 gene (PDB ID: 1UK0). The predicted mutant protein structure’s energy minimization was performed using ANOLEA (Atomic Non-Local Environment Assessment), based on energy calculations on a protein sequence, and checking the Non-Local Environment (NLE) of each heavy atom in the molecule [34]. The energy of each Pairwise interaction was performed using a distance-dependent knowledge-based mean force potential as well as through energy calculations at the atomic level within the protein structure. The calculations were performed on the non-local interactions within all the heavy atoms of amino acids and depending on the energy calculations for each amino acid. High-energy zones (HEZs) indicate fault or interaction zones within a proteins. The 3D structure obtained was based on the high-energy amino acids within the protein. The mutant residue energy calculations were also performed on “chain A” domain of PARP1. The calculated energy shows protein structure stability criteria, displaying general interpretation of differences at the structure level.

### Protein stability and functional effect analysis

Prediction of protein mutant stability adjustments (PoPMusic v2.1) [35] was used to check the stability of the PARP1 Val762Ala based on binary classifications (impact/neutral) scores. The results were based on the selected ΔΔG values in kcal/mol of the predicted PARP1 Val762Ala structure to examine the modification in folding free energy after mutation (ΔΔG). The stability prediction is primarily a comparative modeling procedure where mutated residue is specified, in place of wild type amino acid. The results showed key information about four aspects of the mutation i.e. disease inflicting, disease relation unknown, observed function altering, and random. 

### The functional and structural impact of the mutant residue on PARP1

The consequence of mutant SNP at the structural level was performed to understand its effect on the protein activity. The structural impact of Val762Ala mutation was carried out using Have yOur Protein Defined (HOPE) program [36]. The details of contacts like metal, DNA, hydrogen bonds, ionic interactions was also generated and examined whether the mutation had any impact on essential contact, structural areas together with motifs, domains, trans-membrane domains etc. Distributed Annotation (DAS) server was used to get annotation information regarding transmembrane regions, accessibilities, secondary structure and phosphorylation sites of the actual protein structure [37]. 

### Quantitative RT-PCR

A 20 mg fresh frozen sample was precisely collected from each patient for RNA isolation. RNA extraction was carried out using Qiagen RNeasy Mini Kit following the manufacturer's instructions with DNase treatment (Qiagen). RNA concentration and quality was analyzed with Agilent 2100 Bio-analyzer (Agilent Technologies, Palo Alto, CA). cDNA from RNA was synthesized using the High Capacity cDNA Archive Kit (Applied Biosystems, Foster City, CA) following the manufacturer's instructions.The resulting cDNA was then subjected to real-time quantitative PCR for evaluation of the relative mRNA levels of PARP1 and GAPDH (glyceraldehyde-3-phosphate dehydrogenase, as an internal control) with the following primers: PARP1: forward:5’- GAGTCGGCGATCTTGGACC -3’, and reverse: 5’- TGACCCGAGCATTCCTCG -3’; GAPDH forward: 5′-AGGTGAAGGTCGGAGTCA-3′, and reverse: 5′-GGTCATTGATGGCAACAA-3′ [38]. Gene-specific amplification was performed using an ABI 7900HT real-time PCR system (Life Technologies, Carlsbad, California, USA) with a 15 µl PCR mix containing 0.5 µl of cDNA, 7.5 µl of 2 x SYBR Green master mix (Invitrogen, Carlsbad, California, USA), and 200 nM of the appropriate oligonucleotide primers. The mix was preheated at 95°C (10 min) and then amplified at 95°C (30 sec) and 60°C (1 min) for 45 cycles. The resolution curve was measured at 95°C for 15 sec, 60°C for 15 sec and 95°C for 15 sec. The Ct (threshold cycle) value of each sample was calculated from the threshold cycles with the instrument’s software (SDS 2.3), and the relative expression of PARP1 mRNA was normalized to the GAPDH value. Data were analyzed using the comparative threshold cycle (2^-ΔCT^) method.

### Statistical analysis

Genotypic and allelic frequencies were computed and checked for deviation from Hardy—Weinberg equilibrium (http://ihg2.helmholtz-muenchen.de/cgi-bin/hw/hwa1.pl). Case-control and other genetic comparisons were performed using the chi-square test and allelic odds ratios (OR), and 95% confidence intervals (CIs) were calculated with Fisher’s exact test (two-tailed). Statistical analysis was performed using SPSS (Statistical Package for the Social Sciences) 16.0 software for Windows. We considered p values <0.05 as significant.

## Results

A total of 99 breast cancer (BR) cases and 96 healthy controls were included in this case control study. The clinical characteristics of breast cancer cases and healthy controls are provided in Table 1. Of 99 confirmed cases of breast cancer, 53 were estrogen receptor positive (ER+), and 43 were estrogen receptor negative (ER-); 49 were progesterone positive (PR+), and 41 were progesterone negative (PR-); and 38 were human epidermal growth factor receptor positive (HER2+), and 52 were human epidermal growth factor receptor negative (HER2-) (Table 1). All the genotypic distributions were consistent with Hardy–Weinberg equilibrium (Table 2). The homozygous ancestral allele was used as a reference to determine the odds of acquiring breast cancer in relation to the other two genotypes. 

**Table 2:** Distribution of genotypes and allele frequencies on PARP1 gene loci among Saudi breast cancer patients and controls.

**Genotype**	**Cases**	**HWE P-value**	**Controls**	**HWE P-value**
rs1136410 (Val762Ala)		0.02674		0.16949
Val/Val (wild)	65 (0.66)		75 (0.78)	
Val/Ala	27 (0.27)		20 (0.21)	
Ala/Ala (variant)	7 (0.07)		1 (0.01)	

In the present study, we found a significant variation in the distribution of PARP1 rs1136410 genotypes between breast cancer cases and the matched healthy controls (p > 0.05). As shown in Table 2 the frequency of PARP1 rs1136410 genotypes in breast cancer cases were 65 (0.66), 27 (0.27), and 7 (0.07) respectively, whereas as in healthy controls the frequencies were 75 (0.78), 20 (0.21), and 1 (0.01) respectively. The homozygous variant Ala762Ala (OR= 8.077, χ^2^ =5.11, p= 0.02379) in breast cancer patients showing significant risk when compared to healthy individuals (Table 3). The frequency of rs1136410 (Ala) variant genotype was higher in breast cancer cases (0.2) when compared to healthy controls (0.11) (OR=2.018, χ^2^ = 6.16, p = 0.0131) (Table 3). 

**Table 3:** Genotype Frequencies of PARP1 Gene Polymorphism in Breast Cancer Cases and Controls.

**Genotype**	**Cases**	**Controls**	**OR**	**95% CI**	**Χ^2^**	**p - value**
rs1136410 (Val > Ala)						
Val/Val (wild)	65 (0.66)	75 (0.78)	Ref			
Val/Ala	27 (0.27)	20 (0.21)	1.558	0.8-3.034	1.71	0.19110
Ala/Ala (variant)	7 (0.07)	1 (0.01)	8.077	0.97-67.4	5.11	0.02379
Val/Ala + Ala/Ala	32 (0.34)	21 (0.22)	1.868	0.99-3.53	3.74	0.05307
Val	153 (0.8)	206 (0.89)	Ref			
Ala	39 (0.2)	22 (0.11)	2.018	1.15-3.54	6.16	0.01310

In Saudi breast cancer patients, the median age of onset of breast cancer is 48 years, which is considerably lower than the 62 years observed in the United States [1]. To evaluate the association of PARP1 SNP rs1136410 with the age at diagnosis of breast cancer, we stratified the breast cancer cases as ≤ 48 (n = 46) and > 48 (n = 53) years of age. The genotypic distribution of each SNP and the statistical analysis are shown in table 4. Interestingly, in older age group patients (>48 years) homozygous variant allele Ala762Ala was associated with slightly increased risk of breast cancer (OR= 11.029; χ^2^ =6.9, p= 0.0086) (Table 4). The frequency of rs1136410 (Ala) variant genotype in ER+ group was higher in breast cancer cases (0.23) when compared to healthy controls (0.11) (OR=2.262, χ^2^ = 6.54, p = 0.01053) (Table 4). 

**Table 4:** Genotype Frequencies of PARP1 Gene Polymorphism in Breast Cancer Cases below 48 and above 48 years.

**Genotype**	**Case Parameter**	**Control**	**OR**	**95% CI**	**Χ^2^**	**p- value**
	**< 48 Y**					
Val/Val (wild)	36 (0.65)	42 (0.79)	Ref			
Val/Ala	17 (0.31)	10 (0.19)	1.983	0.807-4.87	2.27	0.13215
Ala/Ala (variant)	2 (0.4)	1 (0.02)	2.333	0.20-26.80	0.49	0.48478
Val/Ala + Ala/Ala	19 (0.35)	11 (0.21)	2.015	0.848-4.79	2.56	0.10969
Val	89 (0.81)	94 (0.89)	Ref			
Ala	21 (0.19)	12 (0.11)	1.848	0.859-3.97	2.52	0.11256
	**> 48 Y**					
Val/Val (wild)	29 (0.66)	33 (0.27)	Ref			
Val/Ala	10 (0.23)	10 (0.23)	1.138	0.415-3.12	0.06	0.80167
Ala/Ala (variant)	5 (0.1)	0 ()	12.492	0.662-235	5.24	0.02202
Val/Ala + Ala/Ala	15 (0.24)	10 (0.23)	1.707	0.665-4.38	1.25	0.26418
Val	68 (0.77)	76 (0.88)	Ref			
Ala	20 (0.23)	10 (0.12)	2.235	0.978-5.10	3.76	0.05264

We have also assessed the association of breast cancer risk with PARP1 SNP rs1136410 based on the estrogen receptor (ER) status of the patients. The genotype distribution in the ER+ (n = 53) and ER- (n = 43) groups was separately compared with the genotype frequency in the healthy individuals (n = 96) (Table 5). The SNP rs1136410 which showed significant association with an increased risk of breast cancer in the overall study population, exhibited significant association with breast cancer risk in ER+ group at variant allele Ala762Ala (0.11) (OR=11.029, χ^2^ = 6.9, p = 0.00863) when compared with controls (Table 5). This association was not observed in the ER-ve category as well as in the overall study population.

**Table 5:** Genotype Frequencies of PARP1 Gene Polymorphism in Breast Cancer Cases HER positive and HER negative.

**Genotype**	**Case Parameter**	**Control**	**OR**	**95% CI**	**Χ^2^**	**p- value**
	**ER+ve**					
Val/Val (wild)	34 (0.64)	75 (0.78)	Ref			
Val/Ala	14 (0.26)	20 (0.21)	1.544	0.698-3.416	1.16	0.28179
Ala/Ala (variant)	5 (0.10)	1 (0.01)	11.029	1.24-98.05	6.90	0.00863
Val/Ala + Ala/Ala	19 (.36)	21 (0.22)	1.996	0.951-4.188	3.40	0.06538
Val	82 (0.77)	206 (0.89)	Ref			
Ala	24 (0.23)	22 (0.11)	2.262	1.198-4.271	6.54	0.01053
	**ER -ve**					
CC (wild)	31 (0.72)	75 (0.78)	Ref			
CT	10 (0.23)	20 (0.21)	1.210	0.508-2.878	0.19	0.66664
TT (variant)	2 (0.05)	1 (0.01)	4.839	0.42-55.33	1.94	0.16417
CT+TT	12 (0.28)	21 (0.22)	1.382	0.607-3.150	0.60	0.43979
C	72 (0.84)	206 (0.89)	Ref			
T	14 (0.16)	22 (0.11)	1.503	0.728-3.101	1.22	0.26848
rs1136410 (Val > Ala)	**PR+**					
Val/Val (wild)	32 (0.65)	75 (0.78)	Ref			
Val/Ala	13 (0.27)	20 (0.21)	1.523	0.677-3.43	1.04	0.30763
Ala/Ala (variant)	4 (0.08)	1 (0.01)	9.375	1.008-87.19	5.50	0.01906
Val/Ala + Ala/Ala	17 (35)	21 (0.22)	1.897	0.886-4.064	2.76	0.09686
Val	77 (0.79)	206 (0.89)	Ref			
Ala	21 (0.21)	22 (0.11)	2.107	1.094-4.060	5.11	0.02383
	**PR -ve**					
Val/Val (wild)	28 (0.68)	75 (0.78)	Ref			
Val/Ala	11 (0.27)	20 (0.21)	1.473	0.627-3.46	0.80	0.37246
Ala/Ala (variant)	2 (0.05)	1 (0.01)	5.357	0.467-61.42	2.24	0.13453
Val/Ala + Ala/Ala	13 (0.32)	21 (0.22)	1.658	0.733-3.752	1.49	0.22244
Val	67 (0.82)	206 (0.89)	Ref			
Ala	15 (0.18)	22 (0.11)	1.730	0.847-3.535	2.30	0.12956
rs1136410 (Val > Ala)	HER**+ve**					
Val/Val (wild)	25 (0.66)	75 (0.78)	Ref			
Val/Ala	10 (0.26)	20 (0.21)	1.5	0.620-3.63	0.81	0.36678
Ala/Ala (variant)	3 (0.08)	1 (0.01)	9.0	0.895-90.48	4.89	0.02706
Val/Ala + Ala/Ala	13 (0.34)	21 (0.22)	1.857	0.813-4.245	2.19	0.13911
Val	60 (0.79)	206 (0.89)	Ref			
Ala	16 (0.21)	22 (0.11)	2.061	1.015-4.183	4.12	0.04241
	HER**- ve**					
Val/Val (wild)	35 (0.67)	75 (0.78)	Ref			
Val/Ala	15 (0.29)	20 (0.21)	1.607	0.736-3.508	1.43	0.23141
Ala/Ala (variant)	2 (0.04)	1 (0.01)	4.286	0.376-48.86	1.61	0.20443
Val/Ala + Ala/Ala	17 (0.33)	21 (0.22)	1.735	0.815-3.69	2.07	0.1504
Val	85 (0.82)	206 (0.89)	Ref			
Ala	19 (0.18)	22 (0.11)	1.727	0.887-3.365	2.62	0.10536

Association of breast cancer risk with the individual SNPs based on the progesterone receptor (PR) status of the tumors was also analyzed. The genotype distribution in the PR+ (n = 49) and PR- (n = 41) groups was separately compared with the genotype frequency in the group of normal healthy women (n = 96) (Table 5). Interestingly, Ala762Ala homozygous variant allele association with breast cancer risk was observed in the PR+ category in the study population (OR=9.375, χ^2^ = 5.5, p = 0.019)) (Table 5). 

We analyzed the association of breast cancer risk with the individual SNPs based on the human epidermal growth factor receptor 2 (HER2) status of the tumors. The genotype distribution in the HER+ (n = 41) and HER- (n = 57) groups was separately compared with the genotype frequency in the group of normal healthy women (n = 96) (Table 5). PARP-1 Ala762Ala homozygous variant allele (OR= 9.0; χ^2^ =4.89, P= 0.02706) and Ala (OR= 2.061; χ^2^ =4.12, p= 0.04241) allele showed a significantly increased risk in HER2+ group patients (Table 5). 

### Mutant and native PARP1 Protein Structure Comparison

**Figure 1:**
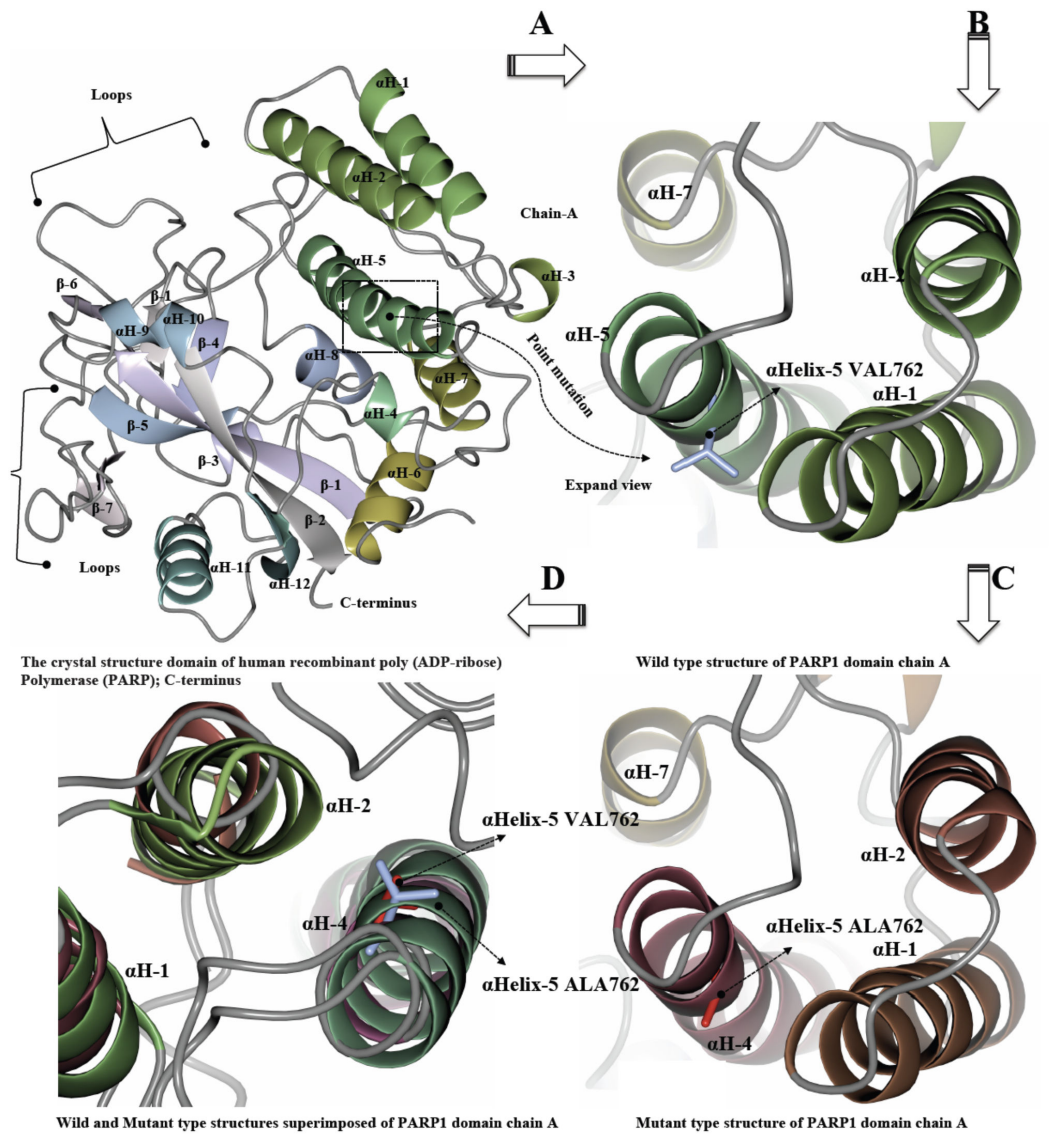
The crystal structure domain of human recombinant poly (ADP-ribose) Polymerase (PARP). (a) Crystal structure domain of the human PARP1 protein structural changes in the regions due to mutation. (b) Wild type structure of PARP1 domain Chain ‘A’ have a point mutation αHelix-5 VAL762 (blue) in a stick representation of the helix region. (c) Mutant type structure of PARP1 domain Chain ‘A’ have a mutation αHelix-5 ALA762 (red) a stick representation of the helix region. (d) Wild and Mutant type structures superimposed of PARP1 domain Chain ‘A’ have wild type residue αHelix-5 VAL762 (red) and mutant residue αHelix-5 ALA762 (blue). Figures (a-d) were made by using CCP4/QTMG.

**Figure 2:**
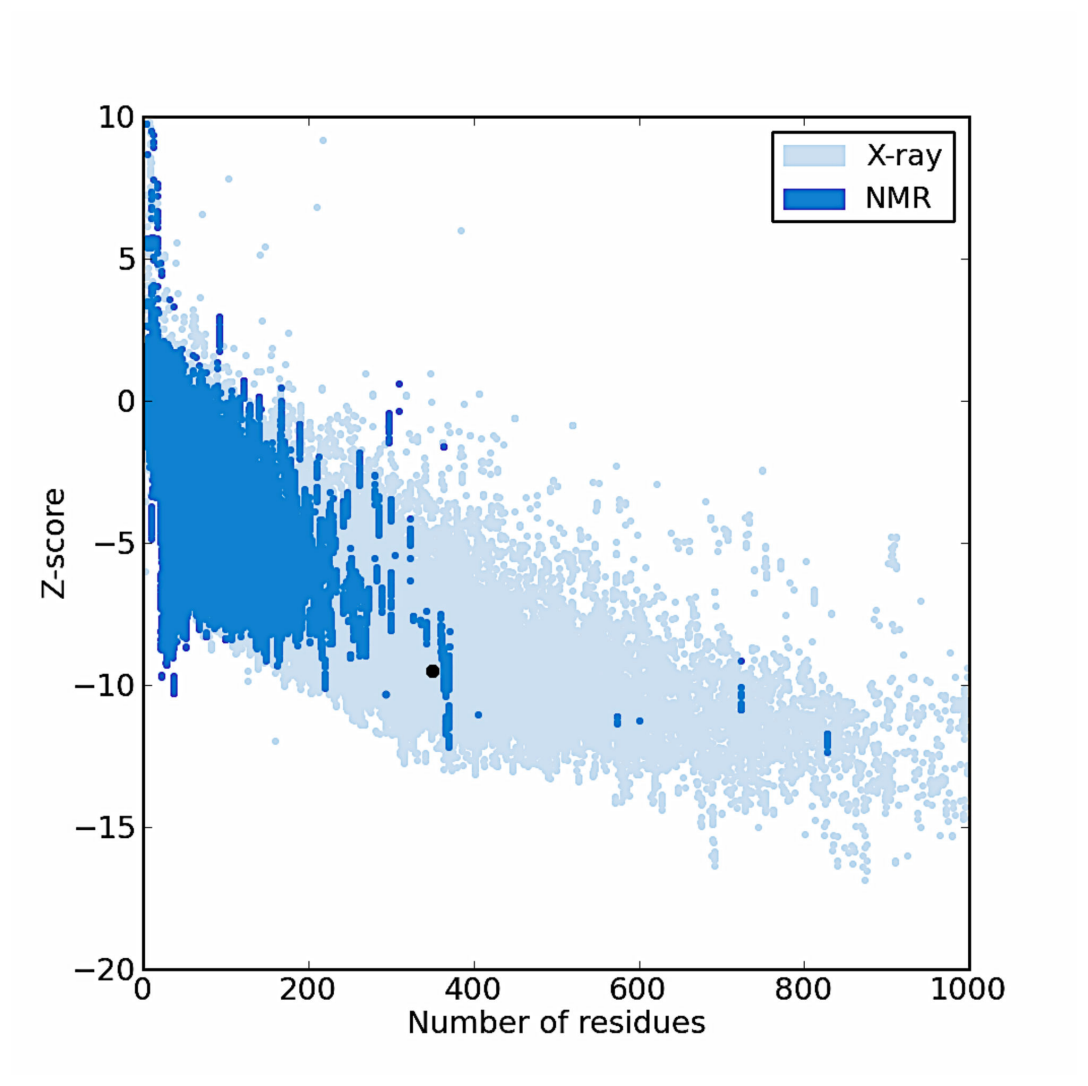
The dbSNP were used to recognize the protein encoded by PARP1 gene (PDB ID: 1uk0) and identified a single mutation residue position. The Z-score, which indicates overall model quality was -9.51 in (black color). The Z-score plot from the different sources (X-ray, and NMR) was distinguished by various colors (X-ray in pale blue and NMR in dark blue color).

**Figure 3:**
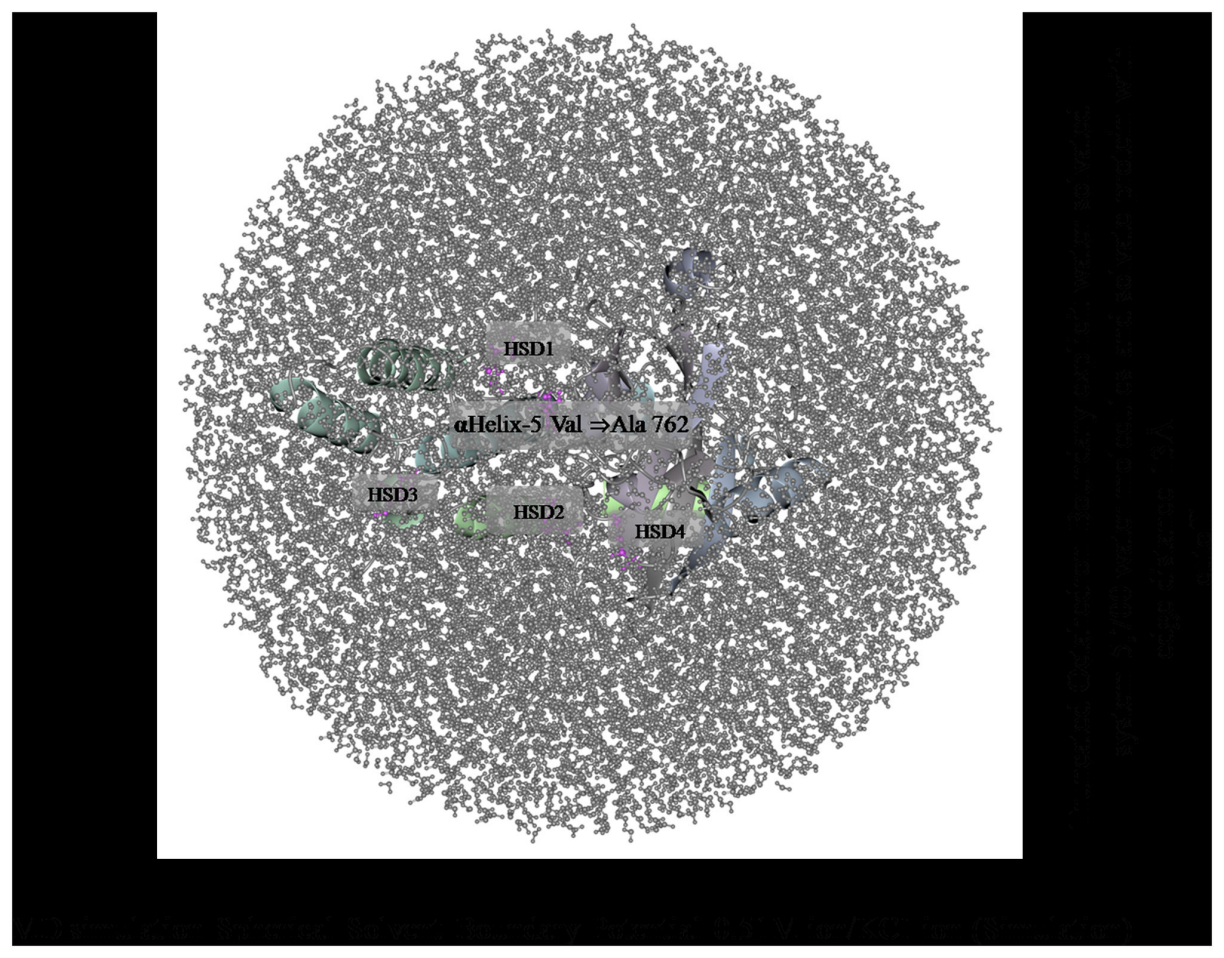
The MD simulation showing truncated octahedron boundary explicit water solvated. The molecular dynamic simulation used in system calculation are, (a) water box surround the entire protein in middle. The visual inspection also allow to identify the side chain of the histidine residue involved in the hydrogen bonding with surrounding molecules and in that case the δ nitrogen of the histidine (HSB;1-4) was protonated residue.

The *PARP1* gene contains the variant allele Val/Ala (rs1136410) at codon position 762 from the crystal structure of catalytic domain of human poly (ADP-ribose) polymerase as shown in (Figure 1). The mutation in the PARP-1 structure was introduced using SWISS-PORT and CCP4 (QtMG) to observe the altered protein structure and to compare it with the native structure. The protein structure validation and quality of the protein structure of native fold were determined by ProSa program. The overall model quality showed-9.51 Z-score (Figure 2). The mutant residue at position 762 was Ala instead of Val within the **α**Helix-5. The iPBA database was used to find similar or partially similar protein folds and to superimpose the two protein structures. The results of the superimposed PARP1 protein domain “Chain A” of the both wild and mutant showed structural alignment with a good RMSD score i.e. 1.50 A°. The results showed that out of 354 residues 346 aligned (97.74% fraction aligned). The energy calculation was performed using ANOLEA program. The PARP1 domain before the mutation showed -2.655 E/KT energy (Boltzmann constant) whereas after mutation the energy was -2.222 E/KT. We further analyzed the whole PARP1 domain “Chain A” structure for its energy which was -511 E/KT and after energy minimization it was less than -5.00 E/KT, when the two energies were compared. The CHARMM program was utilized for MD simulations and for analyzing native and mutated structures under solvent conditions. The solvate showed successful aqueous solvent accumulation around the predicted structure. The solvate with octahedral shapes of water box fully fitted to solvate the molecule with edge distance 13.0 (Figure 3). 

### Protein stability alterations upon amino acid substitution

Thermodynamic protein stability changes due to mutation in the PARP1 Val762 Ala were predicted via PopMusic-2.0 based on statistical potentials through solvent accessibility. The results suggest that the amino acid alteration resulted in an excessive-folding free energy (ΔΔG = 1.27 kcal/mol) causing structural destabilizing effects. Moreover, the mutant tertiary structure also showed significant perturbations due to protein folding in the mutated region between predicted and measured stability changes.

### Impact of mutation on structural and functional effects

The result of mutation of Val to Ala at position 762 of **α**Helix-5 showed that the backbones were same for each amino acid. However, the amino acids differ in their specific size, charge, and hydrophobicity-value, hence the wild-type residue and mutated one differ for these properties. As a result the mutant residue was smaller than the wild-type residue. The results showed that the mutation will alter following features such as contacts made by the mutated residue and structural domains where the residue was located. This mutant residue was part of an interpro domain named "Poly (ADP-ribose) polymerase, regulatory domain" (IPR004102) and Gene-Ontology (GO) annotation indicated its function in NAD^+^ ADP-ribosyltransferase activity (GO: 0003950). Generally, these GO annotations pointed out that the domain plays a role in transferase activity (GO: 0016740) and the mutated residue where it is located in the domain is important for the protein activity. 

### Comparison of PARP1 gene expression between the different stages of breast cancer

**Figure 4:**
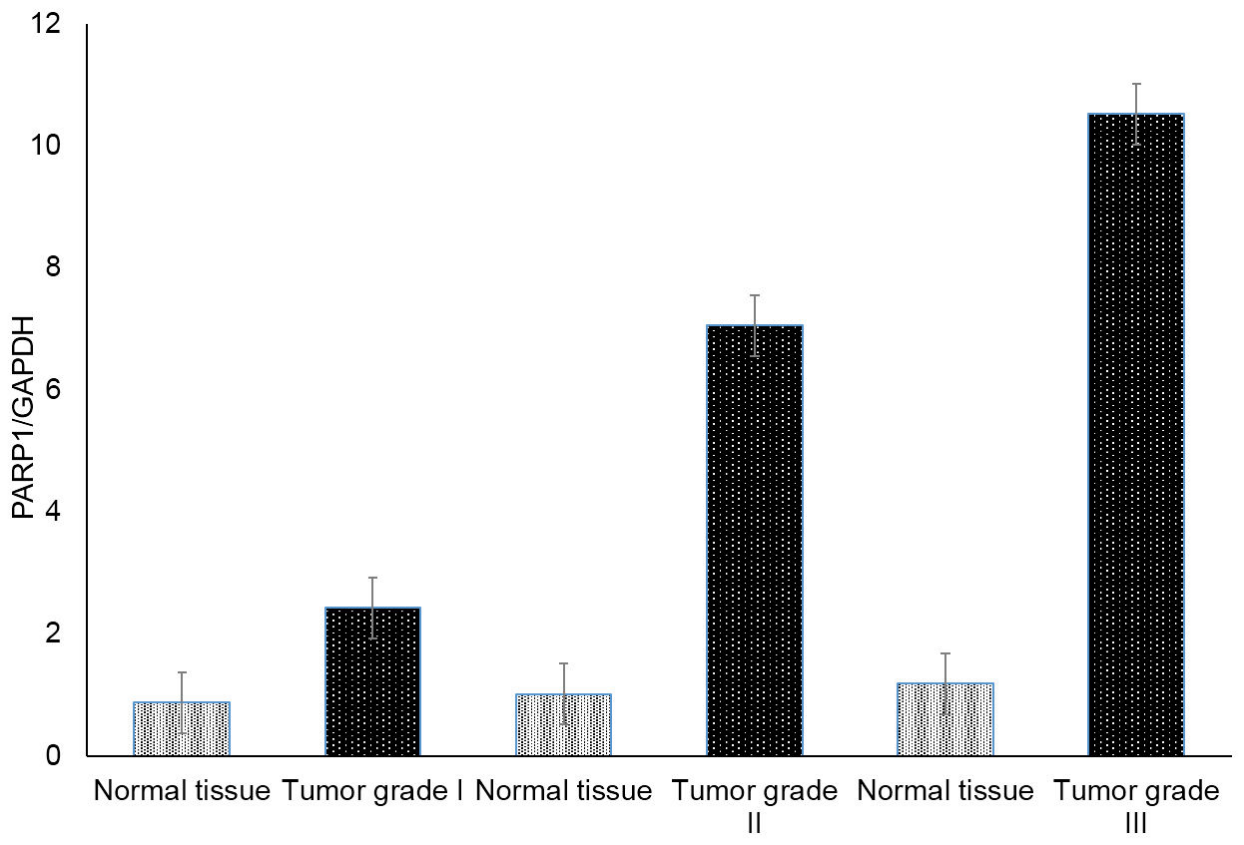
Relative PARP1 mRNA expression in different grades of breast cancer. (Ct values are plotted ±SEM).

Gene expression patterns were quantified to investigate the PARP1 expression levels in breast cancer samples and controls. The expression level of the PARP1 gene was also correlated with tumor grade. The resulting information is provided in Figure 4. PARP1 was found to be expressed in both normal/benign breast tissue and breast cancer specimens. Expression of PARP1 is significantly higher in breast cancer compared to 'normal'/benign breast tissue samples (*P* < 0.001). There was a significant trend for expression levels to increase with the tumor grade in breast cancer patients.

## Discussion

DNA repair mechanisms play a major role in protecting cells against DNA damage and carcinogenesis. Any genetic defects in DNA repair mechanisms will lead to human cancer [7]. By repairing DNA damage and maintaining genetic stability, PARP-1 is playing a key role in prevention of carcinogenesis [11]. PARP-1 catalyze polymerization of ADP-ribose from NAD^+^ to nuclear proteins, such as histones, X-ray repair cross-complementing factor-1, NFkB, p53 and PARP-1 itself [11,12,39]. One of the key variant in PARP-1 gene is Val762Ala (rs1136410) polymorphism. The risk allele changes the amino acid Valine to Alanine at position 762. The PARP-1 Val762Ala (rs1136410) polymorphism has been implicated in cancer susceptibility. It is reported to be associated with increased risk of prostate cancer, esophageal squamous cell carcinoma, smoking-related lung cancer and gastric cardia cancer [19,20,23,40].

In the present case–control study we observed a significant association between the PARP1 Val762Ala polymorphism and the risk of breast cancer in Saudi population. PARP1 Val762Ala showed significant risk at risk at Ala/Ala, Val/Ala + Ala/Ala genotypes and at Ala allele in overall study (Table 3). We have also observed that PARP1 Val762Ala is associated with the Age, PR status, HER2 status and breast cancer susceptibility in Saudi population (Table 4 and 5). PARP1 Val762Ala also showed increased risk of breast cancer at Val/Ala allele in older age and at Ala allele in younger age, PR positive and HER2 positive patients groups (Tables 4 and 5). Interestingly PARP1 Val762Ala didn’t showed any association with ER status. ER, PR and HER status association with breast cancer was not yet reported in this study population. 

In this study, we found for the first time that the PARP-1 Ala762Ala (rs1136410) genotype significantly contributes to breast cancer susceptibility in Saudi population, which further extend the important role of PARP-1 in carcinogenesis. The increased risk of breast cancer in subjects with the PARP-1 Ala762Ala (rs1136410) genotype is likely attributable to the reduction of PARP-1 activity. Until now, studies that investigated associations between the PARP-1 V762A polymorphism and cancer risk have yielded inconsistent results [24]. In a recent meta-analysis study by [24] reported that Asian populations have higher risk of cancer and Caucasians have decreased risk of cancer with PARP-1 Ala762Ala variant. The reason for these differences is currently unclear, but might be explained by ethnical and cultural differences, leading to exposition to different risk factors.

In the present study we have also predicted the effect of Ala762Ala variant on PARP1 molecular structure. The standard molecular dynamics method was applied for analyzing wild and mutant residues using simulations in explicit solvent and examining the differences in dynamics and stability of the PARP1 protein due to 762Ala variation. The energy minimizations studies of the wild type protein (Val762) and the mutant type (762Ala) structures showed that the total energy of the wild type protein structure after energy minimization was - 5.00 E/KT, which was -511 E/KT prior to energy minimization. The mutant PARP1 stability based on thermodynamic changes due to 762Ala was also observed using linear mixture of statistical potentials. We have observed that 762Ala variant is causing structural destabilizing effects. Further MD simulations were carried out to observe the mutation under explicit solvent environment. The solvator was also implemented to create a realistic aqueous solvent environment around the A chain of the PARP1 protein to determine the dimension of system with octahedral shapes of water box (Figure 3). Functional and structural studies of the wild type and mutant PARP1 demonstrated several multimer contacts including the one associated with NAD^+^ ADP-ribosyltransferase activity (GO:0003950) and molecular function (GO:0016740). PARP1 Val762Ala was present in "Poly (ADP-ribose) polymerase, regulatory domain" which is responsible for its prime activity and was shown to be part of NAD^+^ ADP-ribosyltransferase activity (IPR004102). PARP1 Val762Ala residue was located on the surface of helix and which will be in contact with other domains. However, contacts with other molecules or domains may probably be affected by 762Ala mutation. Similar to the report form Ye et al. [11] which assumed the crystal structure of the catalytic domain of human PARP-1 reveals that 762Val is located in the fifth helix of the PARP-1 N-terminal regulatory sub domain, facing the pocket of the active site [11,41]. Our prediction studies shows that 762Ala is located on the surface and it may be affecting the activity with other domains (Figure 1). 

Recent studies by Lockett et al. [19] reported that the PARP-1 activity with 762Ala is decreased compared to the PARP-1 762Val, and the PARP-1 Ala762Ala genotype is associated with an increased risk for prostate cancer in Caucasian subjects [2004]. Thus, 762Val-to-Ala substitution associated with reduced poly(ADP-ribosyl)ation activity, decreases cellular repair function and therefore causes genome instability, leading to cervical carcinoma susceptibility. Milani et al. [42] reported that PARP1 contains contain functional regulatory SNPs in their promoter regions and SNP rs1136410 expression level is effected by allelic imbalance in cancer cells. Our results suggest that, despite the strong biological plausibility, PARP1 is a susceptibility locus for breast cancer in Saudi population. To the best of our knowledge, this is the first report that deals with the functional and structural analysis of breast cancer associated rs1136410 (Val762Ala) through MD simulations. 

Our study has some strengths: patients and controls came from the same geographical area; genotyping errors were avoided using duplicate samples; markers were tested to assure a true association. For the association testing, we also considered multiple genetic models, adjusted our analyses for possible confounders (age at study), and stratified our sample for multiple variables (age at study, ER, PR, HER status) to explore possible effect modifications.

In the present study on gene expression profile, mean PARP1 expression was significantly higher in breast cancer relative to normal breast tissue. In a recent meta-analysis carried out in a large public retrospective gene expression dataset, revealed that PARP-1 messenger RNA (mRNA) was overexpressed in breast cancer [43]. Our results on PARP1 expression levels match with their findings. PARP-1 overexpression was significantly associated with tumor grade. PARP1 expression levels were significantly altered in grade II and III tissues. These results reveals that PARP1 expression levels are increasing with tumor progression. As the sample size of this study is not sufficiently large and is restricted to Saudi population, the present data should be validated in larger samples and in other ethnic groups.

In summary, our study shows for the first time a significant association between the PARP-1 Val762Ala (rs1136410) genotype and increased risk of breast carcinoma in Saudi patients. These findings suggest that PARP-1 Val762Ala may modulate the occurrence of PARP1 mutations and contribute to breast carcinogenesis. Our findings suggest that PARP-1 Val762Ala variant may play an important role in the development of breast carcinoma. Despite our data supports for a clear association between PARP1 and breast cancer in Saudi population and PARP1 gene plays a major role in the susceptibility to the disease. Here we provide evidence for the first time in-silico structural implications of non-synonymous, breast cancer disease-associated variant Val762Ala via molecular dynamic (MD) simulation. Additional functional as well as association studies investigating gene-gene interactions are required to elucidate this issue using more number of samples.
