# Correction: Association between PARP-1 V762A Polymorphism and Breast Cancer Susceptibility in Saudi Population

**DOI:** 10.1371/journal.pone.0092360

**Published:** 2014-03-07

**Authors:** The PLOS ONE Staff

The third author's name is spelled incorrectly. The correct name is: Zainularifeen Abduljaleel. The correct citation is:

Alanazi M, Pathan AAK, Abduljaleel Z, Shaik JP, Alabdulkarim HA, et al. (2013) Association between PARP-1 V762A Polymorphism and Breast Cancer Susceptibility in Saudi Population. PLoS ONE 8(12): e85541. doi:10.1371/journal.pone.0085541

There is also an error in the second sentence of the Materials and Methods section. Patients included in the study had breast cancer, not cardiovascular disease. The correct sentence reads: These encompassed 99 patients with breast cancer and 96 healthy controls.
